# FINANCIAL AND TIME HELP FROM ADULT CHILDREN DURING THE COVID-19 PANDEMIC

**DOI:** 10.1093/geroni/igac059.224

**Published:** 2022-12-20

**Authors:** Emily Wiemers, I-Fen Lin, Janecca Chin, Anna Strauss

**Affiliations:** Syracuse University, Syracuse, New York, United States; Bowling Green State University, Bowling Green, Ohio, United States; Bowling Green State University, Bowling Green, Ohio, United States; Syracuse University, Syracuse, New York, United States

## Abstract

Pandemic-induced challenges to health and economic well-being for older adults likely increased the need for help from adult children, disrupted the help children provided for needs unrelated to COVID-19, and changed interactions among time help, financial help, and shared housing. This paper uses data from the Health and Retirement Study Core and COVID-19 Module to assess whether adult children’s transfers of time, money, and coresidence with parents responded to the pandemic-related challenges older adults faced. Because of unequal health and economic impacts of the pandemic, non-White and less-educated older adults, and those living in areas hard-hit by the pandemic may have been less likely to receive help from family members, their children may have been less able to substitute financial support for time or in-kind help, and older adults may have experienced greater disruptions in existing help arrangements so we examine differences by socioeconomic status, race-ethnicity, and local pandemic severity.

